# The Predictive Value of Fragmented QRS for Cardiovascular Events in Acute Myocardial Infarction: A Systematic Review and Meta-Analysis

**DOI:** 10.3389/fphys.2020.01027

**Published:** 2020-10-06

**Authors:** Gongming Luo, Qian Li, Jingwei Duan, Yu Peng, Zheng Zhang

**Affiliations:** ^1^The First School of Clinical Medicine, Lanzhou University, Lanzhou, China; ^2^Heart Center, the First Hospital of Lanzhou University, Lanzhou, China; ^3^Gansu Key Laboratory of Cardiovascular Disease, Lanzhou, China

**Keywords:** cardiovascular events, acute myocardial infarction, fragmented QRS, electrocardiogram, mortality

## Abstract

**Objective:** Fragmented QRS (fQRS) have been reported as a predictor of major adverse cardiac events (MACE) and mortality in several studies on cardiovascular disease. However, most studies have yielded discrepant results. This study aimed to explore the correlation between fQRS and cardiovascular events in patients with acute myocardial infarction (AMI) during their hospital stay and follow-up period, and the predictive value of fQRS in the prognosis of AMI.

**Methods:** We searched for relevant studies in four databases, Medline, Embase, PubMed, and the Cochrane Library from January 2010 to March 2020. Our initial search yielded 585 articles. Of these, we screened 19 studies, and finally included a total of 6,914 patients in this analysis, comparing death events or MACE in AMI patients with or without fQRS.

**Results:** Fragmented QRS was significantly associated with a higher risk of in-hospital mortality (OR, 3.97; 95% CI, 2.45–6.44; *p* < 0.00001), long-term mortality (OR, 2.93; 95% CI, 1.76–4.88; *p* < 0.0001), in-hospital MACE (OR, 2.48; 95% CI, 1.62–3.80; *p* < 0.0001), and long-term MACE (OR, 3.81; 95% CI, 2.21–6.57; *p* < 0.00001). In particular, it demonstrated a higher predictive value for in-hospital cardiovascular mortality and long-term all-cause mortality in AMI patients and in-hospital mortality in patients with ST-segment elevation myocardial infarction (STEMI). Moreover, fQRS was also associated with an increased risk of ventricular arrhythmias (OR, 2.76; 95% CI, 1.72–4.43; *p* < 0.0001) and heart failure (OR, 1.65; 95% CI, 1.02–2.66; *p* = 0.04). Fragmented QRS was negatively associated with left ventricular ejection function (LVEF) (MD, −5.47; CI, [−7.03, −3.91]; *p* < 0.00001) and positively associated with a high incidence of coronary artery triple vessel lesions (OR, 2.14; 95% CI, 1.31–3.51; *p* = 0.002) in AMI patients.

**Conclusion:** Fragmented QRS is significantly associated with in-hospital and long-term mortality and MACE in patients with AMI, as well as ventricular arrhythmias and heart failure. Furthermore, it may be a marker of mortality and MACE risk. Moreover, fQRS also indicates a reduced LVEF and a high incidence of coronary artery triple vessel lesions in AMI patients.

**Meta-analysis Registration:** https://www.crd.york.ac.uk/prospero; ID: CRD42020171668.

## Introduction

Acute myocardial infarction (AMI) is a significant cause of death in patients with coronary heart disease (CHD). Although reperfusion therapy is effective in improving the short- and long-term cardiac prognosis of patients with myocardial infarction(MI), risk stratification after a percutaneous coronary intervention (PCI) is still challenging (Wright et al., 2011; Mozaffarian et al., 2015). Moreover, the comorbidities of AMI are associated with an increased incidence of adverse events. For example, the co-existence of diabetes increases the risk of 1-year mortality and adverse cardiovascular events after infarction (Marfella et al., 2018a,b). Furthermore, myocardial function after infarction is a critical indicator for predicting future cardiovascular events. However, the imaging methods used to evaluate myocardial function are expensive and not easy to acquire (Eitel et al., 2014; Stone et al., 2016).

The purpose of established risk stratification tools is to identify individuals at high risk and require more intensive therapy (Vignoli et al., 2019). However, these tools have some limitations in the assessment of emergency admissions for AMI because more clinical and laboratory data are needed. Moreover, these tools do not incorporate the use of new electrocardiographic parameters that represent disorders in the electrical activity of the myocardial cells and provide accurate prognostic information, thereby promoting risk stratification and the evaluation of prognosis beyond traditionally used variables.

Several studies have evaluated the potential clinical usefulness of fragmented QRS (fQRS) to identify patients with poor outcomes. Das et al. found a correlation between fQRS and the presence of a myocardial scar. They reported that fQRS had a higher diagnostic sensitivity and negative predictive value for old MI than a pathological Q wave (Das et al., 2006). During the past decade, extensive clinical studies have demonstrated that fQRS has a predictive value for mortality and major adverse cardiac events (MACE) in patients with AMI. However, the predictive value of fQRS for in-hospital MACE and long-term mortality is still controversial. This systematic review and meta-analysis provide a framework for assessing the value of fQRS in predicting in-hospital and long-term cardiovascular events in AMI patients.

## Methods

### Protocol and Registration

This study was registered in the International Prospective Register of Systematic Reviews (PROSPERO) (CRD42020171668).

### Search Strategy

Two researchers independently searched the Embase, Cochrane-Library, Medline, and PubMed databases to retrieve published studies from January 2010 to March 2020, using a search strategy that included the terms “fragmented QRS,” “acute myocardial infarction,” “STEMI,” “NSTEMI,” “MACE,” “cardiovascular events,” and “mortality.”

### Inclusion and Exclusion Criteria

The inclusion criteria were as follows: (a) cohort studies (prospective or retrospective), cross-sectional studies, or randomized control trials; (b) studies that enrolled patients with recent AMI; (c) studies allocating patients to an fQRS group (experimental group) and a non-fQRS group (control group); (d) the first electrocardiogram recorded on admission or within 48 h of admission; (e) studies reporting in-hospital or long-term mortality/MACE.

The exclusion criteria were as follows: (a) studies including populations with other diseases, such as hypertrophic cardiomyopathy, congenital heart disease, Brugada syndrome, Chagas' disease atrial fibrillation, and renal failure; (b) studies not published in English; (c) studies including patients with unstable angina; (d) publications without full texts or with an unknown date.

### Data Extraction and Preset Endpoints

The following information was obtained from each study: title, first author, year of publication, country, the demographic data of participants, the method used to identify cases and controls, the prevalence of fQRS, the technique used to diagnose AMI, and main conclusions. The prespecified outcomes were mortality and MACE in-hospital or during follow-up.

### Definition

fQRS was defined as the presence of a new R wave (R0) or notching in the nadir of the S wave, or the presence of >1 R0 (fragmentation) in 2 contiguous leads, corresponding to a major coronary artery territory. AMI was defined as acute STEMI and NSTEMI. Mortality was defined by all-cause mortality or cardiovascular death. MACE were defined as cardiovascular death, non-fatal myocardial infarction, or non-fatal stroke (Wise et al., 2019). Heart failure was defined as Killip ≥ 2 (Cenko et al., 2019), or definitions from each trial were used (**Table 3**).

### Bias Assessment

The internal validity of studies was assessed using the Quality in Prognosis Studies (QUIPS) tool (Hayden et al., 2013). A Funnel plot and the Egger test were used to test for any potential publication bias.

### Statistical Analysis

Statistical analysis was performed using Review Manager 5.1, State 16, and Meta-Disc 1.4 software. We extracted the baseline data and end events of the experimental and control groups in each study. For the baseline data, the counting data used the chi-square test, and the continuity data used the *t*-test after summarizing the data: the difference was statistically significant with *p* < 0.05. Meta-analysis was performed according to different endpoint events. We also conducted a subgroup analysis to evaluate NSTEMI and STEMI, inclusion criteria, QRS duration, previous MI, and, more importantly, cardiovascular mortality and all-cause mortality, respectively.

The effect size was presented as the odds ratio (OR), likelihood ratio (LR) indicating how many times more (or less) likely a patient experiencing an endpoint is to express fQRS. Since heterogeneity in endpoints from study to study was anticipated, a random effect model was used for the primary analysis, with study as a random variable. Heterogeneity was assessed the *I*^2^ statistic test and its 95% confidence intervals (CI) and the random-effects model was used to account for significant statistical variation. The sensitivity analysis was performed by calculating the number of patients in the trial as a percentage of the total number analyzed, to value the weight of the overall results of the meta-analysis for each study. Meta-regression analysis was used to assess the potential impact of baseline characteristics on heterogeneity.

## Results

### Study Selection

**Figure 1:**
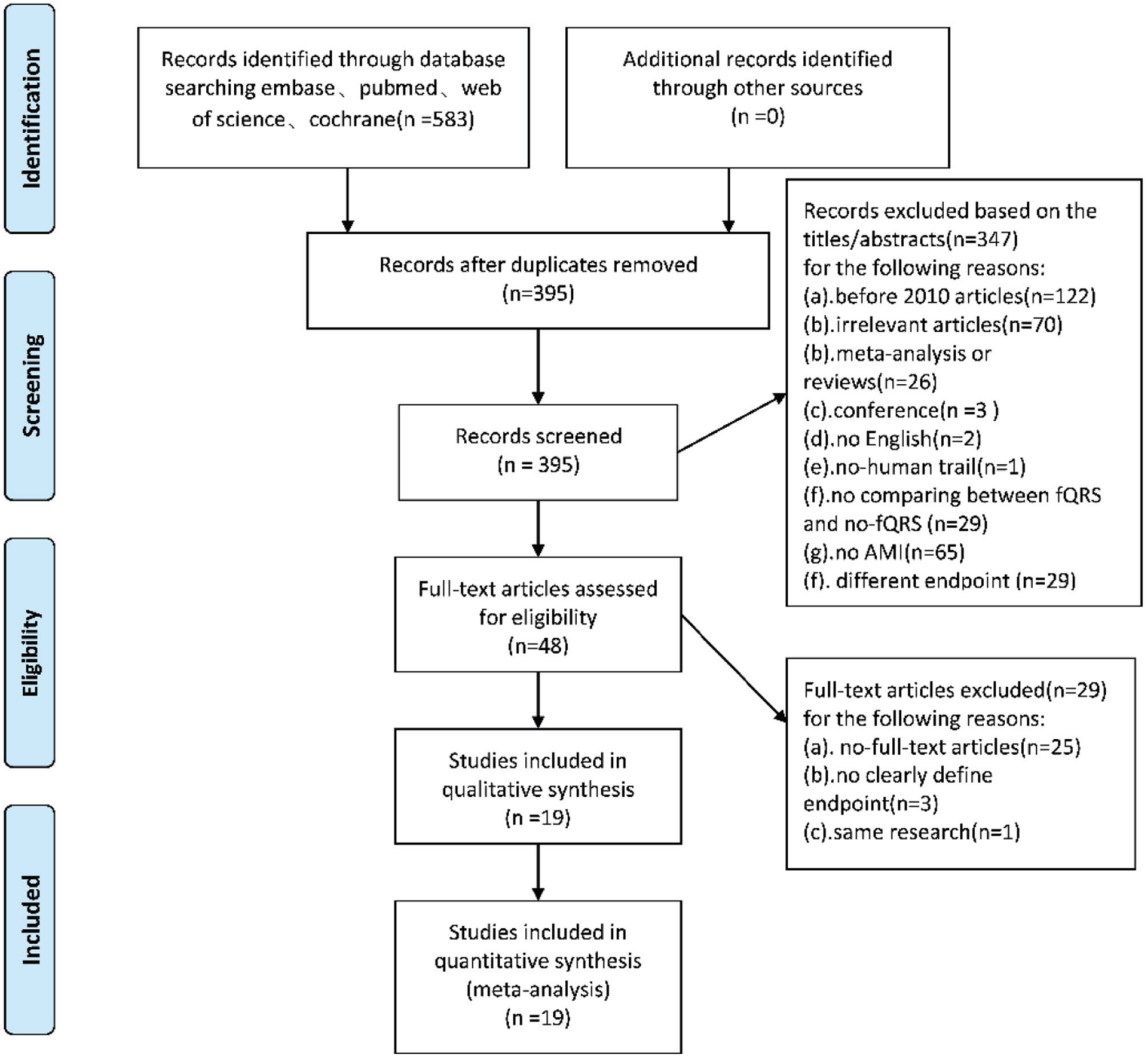
Flow-diagram for the inclusion of studies in this meta-analysis.

Following the above search strategy, initial studies were identified from four online databases. After 188 duplicates were removed, we screened the remaining 395 articles by reading the title and abstract. We excluded 347 articles because they did not meet the inclusion criteria. Out of the remaining 48 articles, 29 were removed after reading the full text. Finally, a total of 19 studies were included in this meta-analysis (Figure 1).

### Quality Assessment of the Included Studies

**Figure 2:**
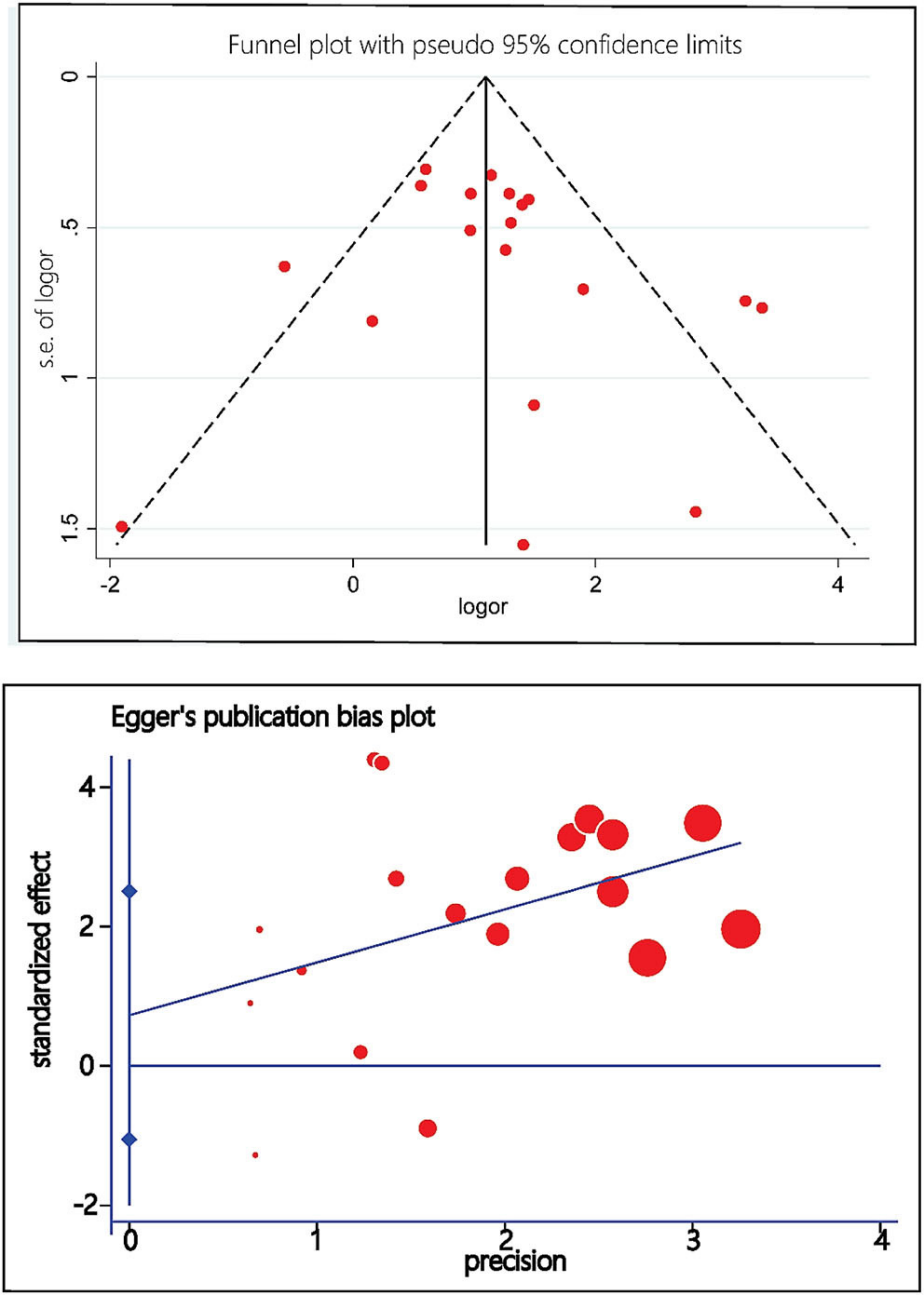
Funnel plot and Egger's test of publication bias.

In general, the methodological quality of the included studies was good, without a high risk of bias. Four studies had different inclusion criteria for fQRS, and therefore the conclusions may be biased compared with other studies (Table 1). Three studies did not give a precise reason for the lack of follow-up, and consequently, the relationship between fQRS and the outcome may be different for completing and non-completing participants (Lorgis et al., 2012; Li et al., 2016; Dural et al., 2019). The funnel plot and Egger test did not suggest evidence of publication bias (Figure 2).

**Table 1:** Quality in prognosis studies.

**Study**	**Study participation**	**Study attrition**	**Prognostic factor**	**Outcome measurement**	**Study confounding**	**Statistical analysis**
			**measurement**			**and reporting**
Ari	Low	Medium	Low	Low	Low	Low
Akgul	Low	Medium	Low	Low	Low	Low
Attachaipanich	Low	Low	Low	Low	Low	Low
Bekler	Low	Medium	Low	Low	Low	Low
Bozbeyogl	Low	Low	Low	Low	Low	Low
Dural	Low	Medium	Medium	Low	Medium	Low
Guo	Low	Medium	Low	Medium	Low	Low
Kurtul	Low	Low	Low	Low	Low	Low
Li	Low	medium	Low	Low	Low	Low
Logis2013	Low	Low	Low	Low	Low	Low
Logis2014	Low	Low	Low	Low	Low	Low
Stavileci	Low	Low	Low	Low	Low	Low
Sheng	Low	Low	Medium	Low	Medium	Medium
Tanriverdi	Low	Low	Low	Medium	Medium	Medium
Umapathy	Medium	Low	Low	Low	Low	Low
Ulsu	Low	Low	Low	Low	Low	Low
Xia	Low	Low	Low	Low	Low	Low
Yildirim	Low	Low	Low	Low	Medium	Medium
Zhao	Low	Low	Low	Low	Low	Low

### Inclusion Criteria for fQRS

Four studies had different inclusion criteria (studies by Akgul, Yildirim, Kurtul, and Umapathy). Patients showing fQRS involving the infarct territory within 48 h of admission were included in the “fQRS group.” Patients who did not develop fQRS even after 48 h of admission were included in the “non-fQRS group.”

Of the remaining 15 studies, the first ECG upon admission showing fQRS was included in the “fQRS group” and the first ECG upon admission without fQRS was included in the “non-fQRS group.”

### Description of Included Studies

Table 2 shows an overview of studies investigating the role of fQRS in cardiovascular events. In terms of baseline data, the incidence of fQRS was about 40%. Most of the participants were male (76%) and received reperfusion interventional therapy (82%). Gender, history of previous MI, previous PCI, and smoking were statistically different between the fQRS and non-fQRS groups. However, hypertension and diabetes were not statistically different between the fQRS and non-fQRS groups while using statistical analysis (Table 3).

**Table 2:** Overview of studies investigating fragmented QRS complex in relation to Cardiovascular events in AMI.

**References**	**Publish**	**First author**	***N***	**Population**	**Type**	**Definition of fQRS**	**Definition of MACE**	**Male**	**PCI**	**Conclusion**	**Follow-up(M)**
Ari et al. (2012)	2011	Ari	85	STEMI	Prospective	The presence of an extra R wave (R1), notched R wave, notched S wave, or more than one R1 wave in 2 contiguous derivations corresponding to the feeding area of one of the major coronary arteries, complete and incomplete BBB were excluded	Cardiac causes mortality, MI, and TVR	85	85	Fragmented QRS may provide great benefits in the evaluation and follow-up of coronary artery disease	6.6 ± 2.3
Akgul et al. (2015)	2014	Akgul	414	STEMI	Prospective	The presence of an extra R wave (R1), notched R wave, notched S wave, or more than one R1 wave in 2 contiguous derivations corresponding to the feeding area of one of the major coronary arteries, complete and incomplete BBB were excluded	Cardiovascular mortality, re-infarction, or TVR (percutaneous or surgical)	328	414	Fragmented QRS was associated with an increased in-hospital cardiovascular mortality, and 1-year all-cause mortality in patients with STEMI who are under primary PCI	12
Attachaipanich and Krittayaphong (2019)	2018	Attachaipanich	452	STEMI	Retrospective	The presence of an extra R wave (R′), notched R wave, notched S wave, or more than one R′, in at least 2 contiguous leads, corresponding to coronary artery territory; a duration of <120 mms, without a typical BBB		344	–	Fragmented QRS complex on admission ECG was an independent predictor of in-hospital life-threatening arrhythmic events in STEMI patients	In-hospital
Bekler et al. (2014)	2014	Bekler	149	NSTEMI	Retrospective	The presence of an extra R' or crochetage wave, notched in the nadir of the S wave, or fragmentation of the RS or QS complexes, in 2 contiguous leads, corresponding to a major coronary artery territory, without a typical BBB pattern (QRS ≥120 ms), or incomplete right BBB	Cardiovascular mortality, re-infarction, or repeat TVR (percutaneous or surgical)	112	39	Fragmented QRS on admission was associated with increased long-term mortality in patients with NSTEMI	18
Bozbeyoglu et al. (2016)	2016	Bozbeyogl	433	NSTEMI	Prospective	The presence of various RSR' patterns, with or without a Q wave and included an additional R wave (R'), notching of the R wave, notching of the downstroke or upstroke of the S wave, or the presence of >1 R', in two contiguous leads corresponding to a major coronary artery territory, no complete or incomplete BBB		290	315	Fragmented QRS may be seen as a cautionary signal for extensive myocardial damage. Therefore, fQRS increases long-term mortality for patients with NSTEMI	12
Dural et al. (2019)	2019	Dural	736	AMI	Prospective	The presence of an additional R wave (R'), R wave or the S wave notching, or the presence of more than one R' wave in two consecutive leads		550	736	Fragmented QRS associated with the presence of adverse events in patients with ACS	12
Guo et al. (2012)	2012	Guo	179	NSTEMI	Retrospective	(1) the presence of various QRS complexes with an additional R', crochetage wave, notching in the nadir of the S wave, or fragmentation of the RS or QS complexes in two contiguous leads corresponding to a major coronary artery territory, (2) absence or presence of the Q wave, and (3) without a typical BBB (QRS ≥120 ms), or incomplete right BBB	Recurrent AMI (STEMI and NSTEMI), unstable angina pectoris, or need for revascularization (PCI or CABG) which did not result in death	113	78	Fragmented QRS was a powerful predictor of decreased survival in NSTEMI. The prognostic importance of fQRS was incremental to clinical and conventional factors	12
Kurtul and Duran (2017)	2017	Kurtul	895	STEMI	Retrospective	The presence of various RSR'patterns with or without a Q wave with an additional R wave, notching of the R wave, notching of the downstroke or upstroke of the S wave, or the presence of >1 additional R wave in 2 contiguous leads corresponding to a major coronary artery territory, without a typical BBB	–	677	895	Fragmented QRS was an independent predictor of postprocedural CIN and in-hospital mortality in STEMI patients	In-hospital
Li et al. (2016)	2016	Li	513	NSTEMI	Retrospective	The presence of an additional R' or crochetage wave, notching in the nadir of the S wave, RS fragmentation, or QS complexes on 2 contiguous leads, without a typical BBB	All-cause mortality, cardiac mortality, recurrent MI, revascularization, recurrent angina and heart failure	399	278	Fragmented QRS complexes were commonly present and an independent predictor of MACE in NSTEMI patients	5.66
Lorgis et al. (2012)	2013	Logis	307	AMI	Retrospective	The presence of various RSR' patterns with or without a Q wave and included an additional R wave, notching of the R wave, notching of the downstroke or upstroke of the S wave, or the presence of >1 additional R in 2 contiguous leads, without complete or incomplete BBB		216	207	Fragmented QRS was an independent predictor of 1-year all-cause mortality after AMI	28
Lorgis et al. (2014)	2014	Logis	209	AMI	Prospective	The presence of various RSR' patterns with or without a Q wave and included an additional R wave, notching of the R wave, notching of the downstroke or upstroke of the S wave, or the presence of > 1 additional R wave in 2 contiguous leads corresponding to a major coronary artery territory, patients with complete or incomplete BBB were excluded	Cardiovascular death or non-fatal recurrent MI	171	171	Fragmented QRS was associated with increased IS, myocardial perfusion abnormalities, decreased LVEF, and increased left heart volumes. fQRS was a reliable marker of infarct size and acute ventricular remodeling	12
Stavileci et al. (2014)	2014	Stavileci	296	STEMI	Retrospective	The presence of notching in R or S waves or extra R waves in the original QRS complex, in at least 2 contiguous leads related to a major coronary artery, a QRS during <120 ms, without a typical BBB	–	226	99	Persistent fQRS was associated with poor prognosis and there was a lack of expected mortality benefit of RPT, particularly that of fibrinolytic therapy, in STEMI patients with fQRS	In-hospital
Sheng et al. (2014)	2014	Sheng	300	AMI	Retrospective	(1) narrow-fQRS(n-fQRS) :Various RSR' patterns with different morphologies of the QRS complexes, with or without the Q wave, in at least 2 contiguous leads, corresponding to a myocardial territory, without BBB(2) wide fQRS (f-wQRS): For a wide QRS wave of BBB and wide QRS complex of premature ventricular contractions (PVCs)	–	–	–	Fragmented QRS could lower the incidence of the cardiovascular events	In-hospital
Tanriverdi et al. (2017)	2017	Tanriverdi	330	STEMI	Retrospective	The presence of various RSR' patterns, with or without Q wave, included an additional R wave (R' prime) or notching of the R wave or S wave, or more than one R' (fragmentation), QRS duration <120 mms, without typical BBB	–	259	330	One or more than one leads with fQRS can be useful when describing the patients under high cardiac risk in acute STEMI	In-hospital
Umapathy et al. (2018)	2018	Umapathy	103	STEMI	Prospective	The presence of an additional R wave (R0) or notching in the nadir of the S wave or the presence of >1 R' (fragmentation) in two contiguous leads, corresponding to a major coronary artery territory, without BBB	All-cause mortality, readmission with ACS or congestive heart failure, or VA	90	102	Fragmented QRS did not predict MACE and LV dysfunction in STEMI patients belonging to Killip class I and II on short term follow-up of 30 days. However, fQRS independently predicted impaired microvascular myocardial reperfusion as assessed by SigmaSTR	1
Uslu et al. (2015)	2014	Ulsu	542	STEMI	Retrospective	The presence of notched R or S waves, or an additional wave like RSR' pattern in the original QRS complex, a duration of <120 ms, without a typical BBB	Cardiovascular mortality, reinfarction, or repeat TVR	435	542	Fragmented QRS reflected the linking between impairment of regional left ventricular systolic function and the presence of severe myocardial injury in STEMI	18
Xia and Feng (2018)	2018	Xia	420	STEMI	Retrospective	Typical RSR' patterns, with or without the Q wave, included a notching of R wave or S wave or an additional R wave, or the presence of more than 1 R prime, a QRS during <120 mms, without a typical BBB	–	242	259	Fragmented QRS was an independent predictor for the adverse cardiac events in STEMI patients undergoing PCI or thrombolysis	In-hospital
Yildirim et al. (2014)	2014	Yildirim	335	STEMI	Prospective	Presence of an additional R wave, or a notch in the tip of the S wave, or more than 1 large R' wave in 2 consecutive derivations	–	270	335	Fragmented QRS and number of fQRS derivations were a significant predictor of in-hospital major cardiac events in STEMI patients	In-hospital
Zhao et al. (2018)	2018	Zhao	216	STEMI	Retrospective	(1) The presence of notched R or S, or the existence of an additional wave—like R S R ' pattern in the original QRS complex; (2) waves with or without a Q wave; (3) a duration of <120 ms; and (4) no accompanying typical BBB	Cardiovascular mortality, reinfarction, advanced heart failure, repeat TVR, VA, atrioventricular block or stroke	161	216	Fragmented QRS was a prognostic marker of impaired regional ventricular systolic function	12

*BBB, bundle branch block; AMI, acute myocardial infarction; STEMI, ST-segment Elevation Myocardial Infarction; NSTEMI, non-ST-segment Elevation Myocardial Infarction; ACS, acute coronary syndrome; GABG, coronary artery bypass surgery; TVR, target vessel revascularization; VA, ventricular arrhythmia*.

**Table 3:** Baseline and procedural characteristics according to types of AMI.

		**fQRS**	**Non-fQRS**	***P*-value**
Past history and	Age(avg ± SD)	56.6 ± 13.7	59.0 ± 12.9	<0.001
clinical features	Male	1,846	3,163	<0.001
	DM^a^	781/2,547(31%)	1,115/3,918(28%)	0.057
	HPT^b^	1,226/2,456(50%)	1,230/3,595(34%)	0.333
	Prior MI^c^ History of CHD^d^ Prior PCI/CABG^e^	143/539(27%) 272/934 202/1,142	155/1,329(12%) 381/1286 174/2,007	<0.001 0.797 <0.001
	Smoking	875/1,650(53%)	1749/2,909(60%)	<0.001

a DM, diabetes mellitus;

b HPT, hypertension;

c prior MI prior myocardial infraction;

d history of CHD history of coronary heart disease;

e *PCI/GABG percutaneous coronary intervention/ coronary-artery bypass grafting*.

### Meta-Analysis

#### Clinical Characteristics

All studies, except those conducted by Sheng et al., Li et al., and Logis et al. reported left ventricular ejection function (LVEF) in study groups. Of the 19 studies, 16 studies reported LVEF, and 13 reported LVEF as mean ± standard deviation. Thirteen studies demonstrated a significant negative correlation between LVEF in patients with fQRS and AMI (MD, −5.47; CI, [−7.03, −3.91]; *p* < 0.00001) with significant heterogeneity (*I*^2^ = 84%). Moreover, seven studies reported an obvious relationship between fQRS and triple vessel lesions (OR, 2.14; 95% CI, 1.31–3.51; *p* = 0.002) with significant heterogeneity (*I*^2^ = 79%).

#### fQRS and Mortality

##### fQRS and in-hospital mortality

**Figure 3:**
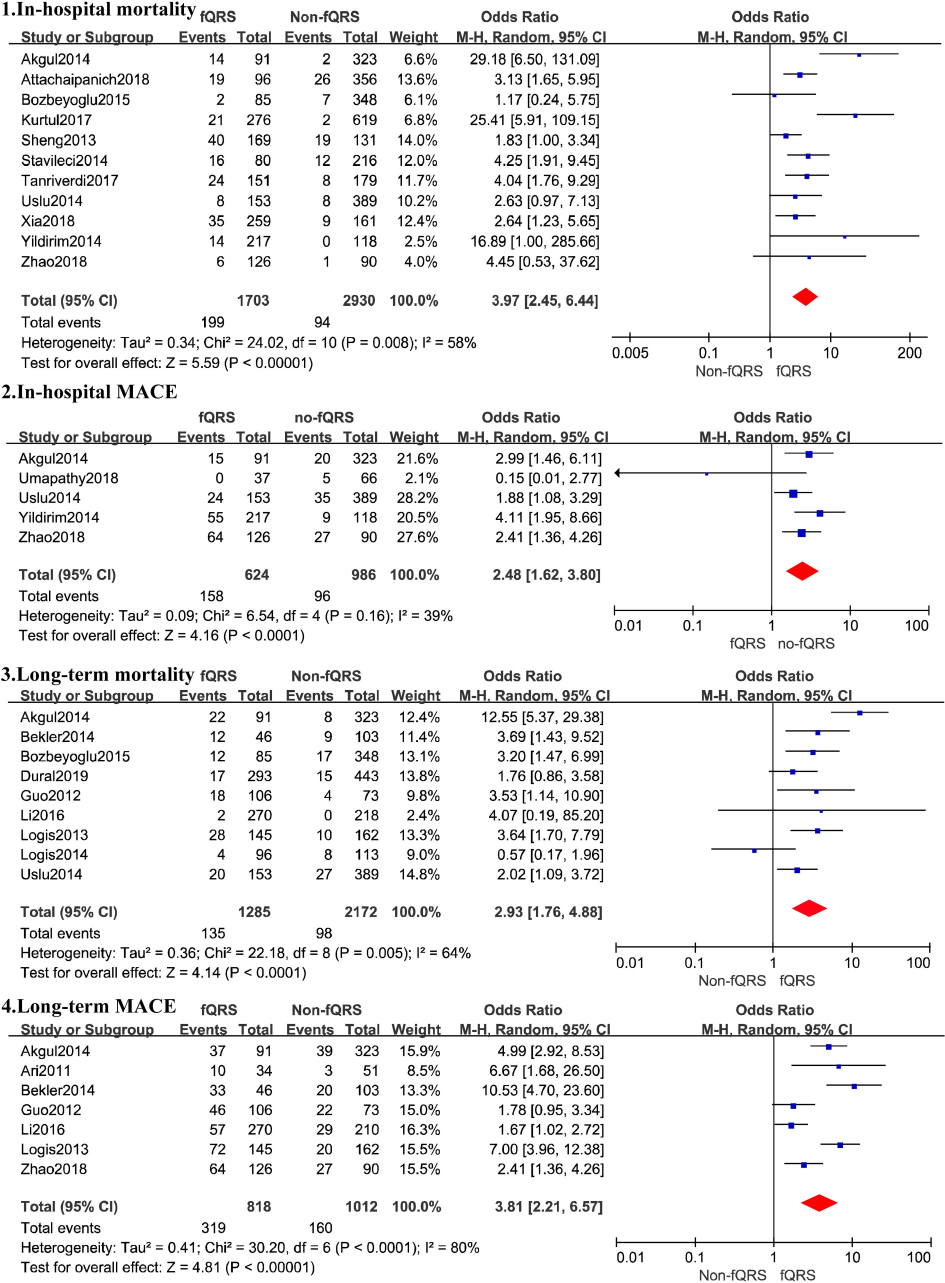
Summary of the odds risk of mortality and MACE in patients with fQRS. The size of the square for each study is proportional to the sample size. Weighting refers to the contribution of each study to the pooled result and was determined using the random effects model (the endpoints reported in the forest plot are the odds risk of in-hospital mortality, in-hospital MACE, long-term mortality, and long-term MACE, respectively).

**Figure 4:**
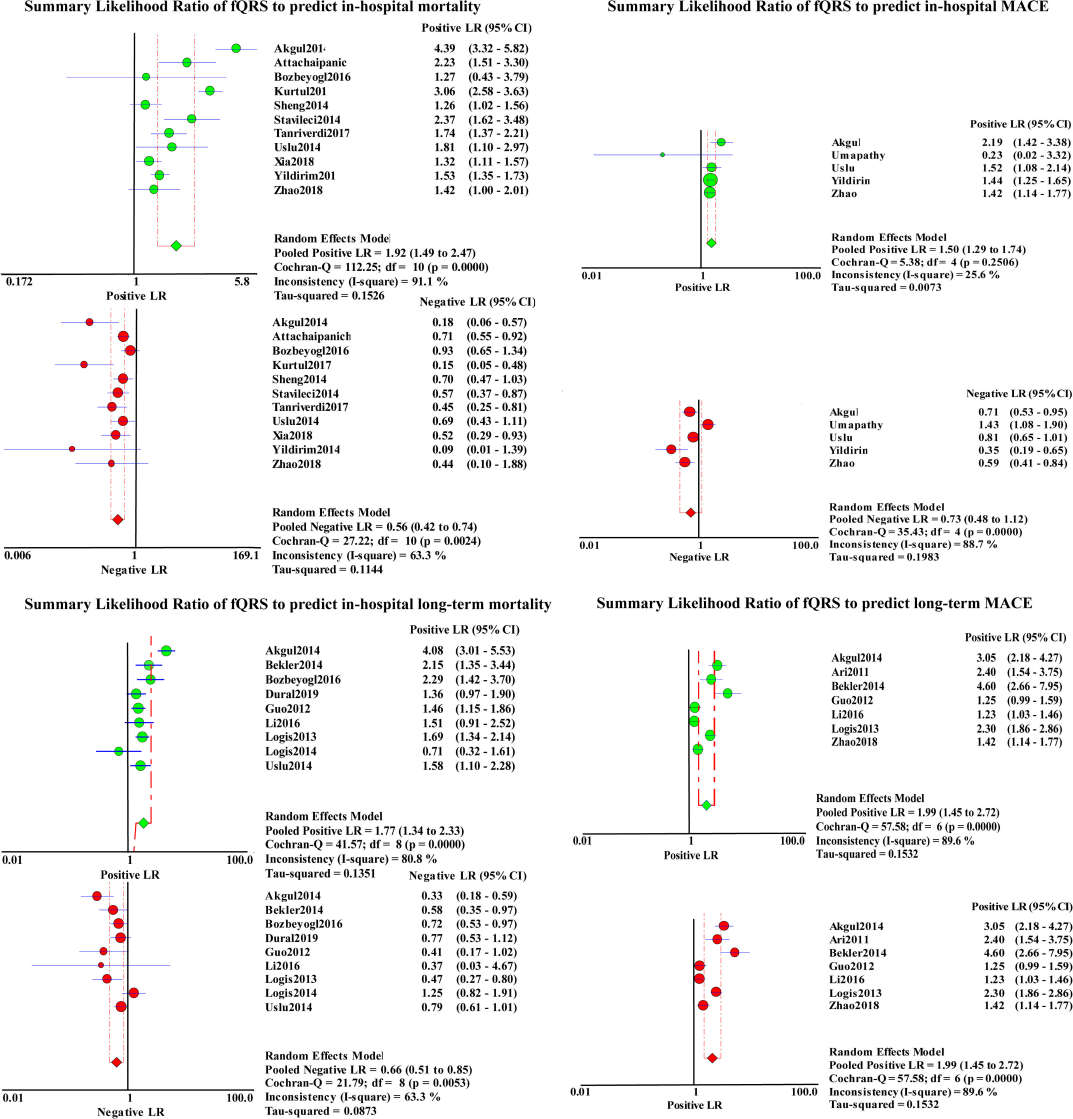
Summary LRs of fQRS to predict in-hospital mortality, in-hospital MACE, long-term mortality, and long-term MACE.

A total of 4,633 patients from 11 studies were included in the analysis of in-hospital mortality (Figure 3). Fragmented QRS was significantly associated with a higher risk of in-hospital mortality (OR, 3.97; 95% CI, 2.45–6.44; *p* < 0.00001) with obvious heterogeneity (*I*^2^ = 58% *p* = 0.008). The summary positive LR was 1.94 (95% CI, 1.49–2.52) and the negative LR was 0.55(95% CI, 0.41–0.75). The positive and negative LRs revealed significant heterogeneity (*I*^2^ = 91.9%, *p* < 0.0001) (*I*^2^ = 67.2%, *p* = 0.0007) (Figure 4). The heterogeneity was verified by identifying three studies by Yildirim et al. (2014), Akgul et al. (2015), and Kurtul and Duran (2017), using meta-regression analysis. According to the fQRS criteria, we divided the patients into two subgroups: (1) the fQRS positive group (*an arbitrary ECG group*), if fQRS appeared in any ECG within 48 h; the OR was 23.32 (95% CI, 6.38–85.20); there was no heterogeneity in the subgroup (*I*^2^ = 0%, *p* = 0.8). (2) the fQRS positive group (*specific time group*), if fQRS was found in the ECG on admission or at the 48th h; the OR was 2.77 (95% CI. 2.06–3.72); there was no heterogeneity in the subgroup (*I*^2^ = 0%, *p* = 0.64). Meanwhile, the heterogeneity was verified by identifying two studies of Akgul et al. and Kurtul et al. using sensitivity analysis. After removing them in the analysis, the overall pooled estimate of OR (2.82, 95% CI, 2.11-3.78) was reduced but removed any significant heterogeneity (*I*^2^ = 0%, *p* = 0.56).

Six studies reported all-cause in-hospital mortality (OR, 3.77; 95% CI, 2.06–6.91; *p* < 0.00001), five reported in-hospital cardiovascular mortality (OR, 4.31; 95% CI, 2.45–6.44; *p* = 0.002), and nine merely calculated the mortality in STEMI patients (OR, 4.92; 95% CI, 2.93–8.28) (Table 4). However, there was significant heterogeneity between these studies before the sensitivity analysis identified three studies by Akgul et al., Kurtul et al., and Yildirim et al. as outliers (Yildirim et al., 2014; Akgul et al., 2015; Kurtul and Duran, 2017). After removing the studies by Kurtul and Duran (2017) and Yildirim et al. (2014) the overall pooled OR (2.32, 95% CI, 1.68–3.21) in the meta-analysis for all-cause in-hospital mortality was reduced, with less heterogeneity between studies (*I*^2^ = 13%, *p* = 0.25). After removing the study by Akgul et al. (2015) alone, the overall pooled OR (2.91, 95% CI 1.73–4.87) in the meta-analysis for in-hospital cardiovascular mortality also somewhat decreased, without any heterogeneity between studies (*I*^2^ = 0%, *p* = 0.60). The pooled OR (3.38 95% CI, 2.40–4.77) in the meta-analysis for the in-hospital mortality in STEMI patients decreased, without any heterogeneity between studies (*I*^2^ = 0%, *p* = 0.86) after removing Akgul et al. and Yildirim et al.'s studies (Yildirim et al., 2014; Akgul et al., 2015). Summary estimates in other subgroups are given in Table 4.

**Table 4:** Results of subgroup analysis and other endpoints.

**Factors**	**Categories**	**No.of studies**	**fQRS group**	**No-fQRS group**	**OR/MD(95%CI)**	**Model used**	**Heterogeneity**	
			**Events/total**	**Events/total**			**I2**	***p*-value**
**Difference endpoints of mortality**								
In-hospital mortality	All-cause	6	153/1,168	64/1,564	3.77 [2.06, 6.91]	Random	63	<0.0001
	Cardiovascular	5	40/409	29/1,276	4.31 [1.75, 10.64]	Random	58	0.002
Long-term mortality	All-cause	4	61/807	38/1,427	4.42 [1.68, 11.60]	Random	75	0.003
	Cardiovascular	8	116/992	79/1,729	3.22 [1.79, 5.80]	Random	66	<0.0001
**Subgroup for main endpoints**								
In-hospital mortality	All	11	185/1,612	92/2,607	3.32 [2.20, 5.01]	Random	58	0.008
	An arbitrary ECG group	3	35/493	2/737	23.32 [6.38, 85.20]	Random	0	0.8
	Specific time group	8	150/1,119	90/1,870	2.77 [2.06, 3.72]	Random	0	0.64
	History of MI^a^	9	154/1,276	84/2,132	3.30 [2.05–5.29]	Random	48	0.05
	First MI	3	59/518	12/1,121	12.91 [3.07–54.19]	Random	75	0.02
	QRS duration <120 mms	9	145/1,317	75/2,681	4.32 [2.57–7.26]	Random	55	0.02
	QRS duration Including≥120 mms	2	54/386	19/249	3.74 [0.43,32.41]	Random	61	0.11
	Retrospective	8	169/1,310	85/2,141	3.39 [2.24, 5.14]	Random	44	0.08
	Perspective	3	30/393	9/789	7.83 [0.80, 76.36]	Random	77	0.01
	NSTEMI^b^	1	2/85	7/348	1.17 [0.24, 5.75]	Random	/	/
	STEMI^c^	8	157/1,449	68/2,451	4.92 [2.93, 8.28]	Random	53	0.03
Long-term mortality	NSTEMI	4	44/507	30/742	3.44 [2.04, 5.80]	Random	0	1
	STEMI	2	42/244	35/712	4.91 [0.82, 29.44]	Random	91	0.0001
	History of MI	7	109/1,098	82/1,736	2.64 [1.92,3.62]	Random	0	0.71
	First MI	2	26/187	16/436	2.77 [0.13–58.56]	Random	94	0.0001
	QRS duration <120 mms	8	133/1,015	98/1,954	2.90 [1.71–4.93]	Random	68	0.002
	QRS duration Including≥120 mms	1	2/270	0/218	4.07 [0.19–85.20]	Random	/	/
	Retrospective	4	52/575	40/783	2.60 [1.64, 4.12]	Random	0	0.67
	Perspective	4	66/417	43/946	3.24 [1.14, 9.22]	Random	82	0.0007
In-hospital MACE	QRS duration Including≥120 mms	1	55/217	9/118	3.32[1.70–6.48]	Random	/	/
	QRS duration <120 mms	4	103/407	87/868	2.19[1.39–3.44]	Random	23	0.27
	History of MI	5	165/632	87/788	2.01 [1.40–2.88]	Random	41	0.15
	First MI	1	15/91	20/323	2.66 [1.42,4.99]	Random	/	/
Long-term MACE	QRS duration Including≥120 mms	1	57/270	29/210	1.67 [1.02,2.72]	Random	/	/
	QRS duration <120 mms	6	262/548	131/802	4.45 [2.56,7.72]	Random	75	0.001
	History of MI	5	210/582	101/527	3.12 [1.62–6.02]	Random	78	0.001
	First MI	2	109/236	59/485	5.85 [3.96–8.64]	Random	0	0.39
**Others endpoints**								
VT/VF^d^	/	6	128/763	95/1,455	2.76 [1.72,4.43]	Random	57	0.04
Heart failure	/	5	158/839	115/1,188	1.65 [1.02,2.66]	Random	64	0.03
TVL^e^	/	7	327/796	287/1,188	2.14 [1.31–3.51]	Random	79	<0.0001
LVEF^f^	/	13			−5.47 [−7.03,−3.91]	Random	84	<0.00001

a MI myocardial infarction;

b NSTEMI Non-ST-segment elevation myocardial infarction;

c STEMI ST-segment elevation myocardial infarction;

d VT/VF Ventricular tachycardia/ventricular fibrillation;

e TVL Triple vessel lesion;

f *LVEF Left ventricular ejection function*.

##### fQRS and Long-Term Mortality

Nine studies representing 3,457 patients with fQRS were included in this meta-analysis for long-term mortality (Figure 3). Of 3,457 patients, 233 died during the mean/median follow up of 5.66–28 months. Mortality was more common in patients with fQRS than in controls (135/1285 vs. 98/2172) (OR, 2.93; 95% CI, 1.76–4.88; *p* < 0.0001), with significant heterogeneity between studies (*I*^2^ =66%; *p* < 0.0001). The pooled positive and negative LRs were 1.77 and 1.66, respectively, with significant heterogeneity in positive LR (*I*^2^ = 80.8%, *p* < 0.0001) and negative LR (*I*^2^ =63.3%, *p* = 0.0053) between studies (Figure 4).

Four studies reported all-cause long-term mortality (OR, 4.22; 95% CI, 1.68–11.60; *p* < 0.003) and eight reported long-term cardiovascular mortality (OR, 3.22; 95% CI, 1.79–5.80; *p* < 0.0001), respectively, both with significant heterogeneity between studies (*I*^2^ = 75%, *p* = 0.003, and *I*^2^ = 66%, *p* < 0.0001, respectively). The sensitivity analysis showed that the study by Akgul et al. (2015) was the outlier, which shared the same cause of heterogeneity in in-hospital mortality. The presence of fQRS was still found to be correlated with long-term mortality risk (OR, 2.40; 95% CI, 1.67–3.47; *p* < 0.00001), all-cause mortality risk (OR, 2.81; 95% CI, 1.40–5.65; p = 0.004), and cardiovascular mortality risk (OR, 2.58; 95% CI, 1.69–3.94; *p* < 0.0001) after ruling out this study from the analysis, with reduced heterogeneity (I^2^ = 25, 37%, and 22%; *p* = 0.23, 0.21, and 0.22, respectively) (Table 4).

The result was not statistically significant (OR, 4.91; 95% CI, 0.82–29.44; *p* = 0.0006; *I*^2^ = 91%) when only patients with STEMI were included (*n* = 956). The fQRS was still found to be correlated with a higher risk of long-term mortality (OR, 3.44; 95% CI, 2.04–5.80; *p* < 0.00001), without heterogeneity (*I*^2^ = 0%, *p* = 1.00) when only patients with NSTEMI were considered (*n* = 1,248). In addition, in the presence of fQRS, whether or not these several studies with obvious heterogeneity are removed. The OR of in-hospital mortality is always higher than that of long-term mortality. In terms of the prediction of in-hospital mortality, the predictive value for in-hospital cardiovascular mortality may be higher than that for all-cause mortality. In terms of long-term mortality, however, the predictive value for all-cause mortality may be higher than that for cardiovascular mortality. Summary estimates in other subgroups are given in Table 4.

#### fQRS and MACE

A total of 1,610 patients with STEMI in 5 studies were included in the analysis for in-hospital MACE (Figure 3). The incidence of MACE was higher in the fQRS group (158/624 vs. 96/986)(OR, 2.48; 95% CI, 1.62–3.80; *p* < 0.0001) than in controls, without significant heterogeneity (*I*^2^ = 39%,). The summary positive LR was 1.50 (95% CI, 1.29–1.74) (Figure 4) without significant heterogeneity (*I*^2^ = 25.6%, *p* = 0.32). The negative LR was 0.73 (95% CI, 0.48–1.12) with significant heterogeneity (*I*^2^ = 86.8%, *p* < 0.0001) (Figure 4).

Outcomes regarding long-term MACE were available in 7 studies (Figure 3). During a mean/median follow up of 5.66–28 months, 479 out of 1,830 patients died. MACE was more common in patients with fQRS (319/818 vs. 160/1012) (OR, 3.81; 95% CI, 2.21–6.57). Significant heterogeneity existed across studies (*I*^2^ = 82%, *p* < 0.0001). The summary positive LR was 1.99 (95% CI, 1.45–2.72) (Figure 4), and the negative LR was 0.54 (95% CI, 0.44–0.68) with significant heterogeneity in the positive LR (*I*^2^ = 89.6%, *p* < 0.0001) and the negative LR(*I*^2^ = 59.2%, *p* = 0.023). Sensitivity analysis and meta-regression analysis failed to identify a single study to account for heterogeneity (Figure 4).

#### fQRS and Other Cardiovascular Events

Five studies including 839 patients and 1,188 patients in the fQRS and the non-fQRS group, respectively, reported heart failure (Table 4). There was an association between fQRS and heart failure (OR, 1.65; 95% CI, 1.02–2.66; *p* = 0.04) with significant heterogeneity (*I*^2^ = 64%, *p* < 0.0001). A total of 6 studies reported VT/VF events (Table 4). Among 2,218 patients, 223 had VT/VF. There was a significant association between fQRS and VT/VF events (OR, 2.76; 95% CI, 1.72–4.43; *p* < 0.0001) with significant heterogeneity (*I*^2^= 57%, *p* = 0.04). Sensitivity analysis and Meta-regression failed to identify the reason for the heterogeneity of the two studies.

#### Subgroup Analysis of Main Endpoints

This meta-analysis conducted a subgroup analysis on the main endpoint events based on whether the QRS duration was included ≥120 mms and whether the patients included in the study had a previous history of MI or PCI. The pooled OR of in-hospital mortality, MACE, and long-term MACE were higher than those in the studies with a previous history of MI. In the subgroup analysis of QRS duration, it was found that the total value of hospital deaths was lower in the group with a QRS duration of included ≥120 mms compared to the group with a QRS duration of <120 mms. The clinical value of other subgroups may be low because there was no statistical significance or small study sample size (Table 4).

## Discussion

The main findings of this meta-analysis are: (1) the fQRS on admission ECG was strongly associated with mortality and MACE in AMI patients during the in-hospital stay and follow-up; (2) fQRS was positively correlated with ventricular arrhythmia (VA) and heart failure in in-hospital patients; (3) fQRS also indicated a decreased LVEF and a high incidence of coronary artery triple vessel lesions in AMI patients.

### The Clinical Significance of fQRS

In this meta-analysis, we found that the presence of fQRS on admission ECG was associated with a nearly 4-fold risk of in-hospital mortality and long-term MACE, and an almost 3-fold risk of MACE and VT/VF in the hospital, and mortality during the follow-up period. Thus, this meta-analysis suggests that fQRS has a definite predictive value for in-hospital and long-term cardiovascular events. In the subgroup analysis, we found that for patients with no previous history of MI and PCI, the emergence of fQRS suggests that the risk of in-hospital death, in-hospital MACE events, and long-term MACE events may be higher, which has not been reported in previous studies. We speculate that the reason for these opposing findings may be that fQRS mainly develops after MI, so the possibility of hemodynamic and electrophysiological instability is higher. Additionally, meta-analyses of previous studies showed that for ischemic cardiomyopathy, the longer the QRS duration, the greater the risk of death. On the contrary, in our meta-analysis, we calculated a higher OR value for studies with a QRS duration of <120 mms (Rosengarten et al., 2015). Unfortunately, the specific reason behind these inconsistencies is not clear; although, it may be related to the inclusion criteria for fQRS.

The comorbidities of MI negatively affect prognosis, especially in patients with diabetes mellitus, which can increase the incidence of adverse events including MACE and death. State of diabetes could cause downregulation of stem endothelial cells implied in regenerative post-STEMI events, and loss of epigenetic regulators implied in intracoronary vessels thrombosis, and prediabetes increases the inflammatory burden in pericoronary adipose tissue (Marfella et al., 2013, 2018a,b; Sardu et al., 2019; D'onofrio et al., 2020). There are often concerns for patients with these complications. However, our meta-analysis did not show a statistical difference in the incidence of diabetes between the fQRS and non-fQRS groups, possibly due to the different mechanisms of cardiovascular events associated with fQRS and diabetes. Moreover, our study found a negative correlation between fQRS and LVEF in patients with MI, which could be the result of multiple vascular diseases and larger infarct area and might also be one of the reasons for poor prognosis (Gungor et al., 2016).

### fQRS and Mortality

Previous studies identified fQRS as a significant predictor of in-hospital mortality in patients with AMI (Das et al., 2007, 2009; Gungor et al., 2016; Attachaipanich and Krittayaphong, 2019). Our meta-analysis suggests that fQRS increases in-hospital and long-term mortality in AMI patients. The underlying mechanism may be related to myocardial scar formation, but there is still a lack of relevant research evidence (Rosengarten et al., 2015). A negative correlation between fQRS and LVEF was demonstrated in our study and previous studies. Moreover, data from this study shows that fQRS increases the risk of triple vessel lesions, indicating patients with advanced coronary artery disease (Thuijs et al., 2019). Previous studies have reported that fQRS is related to the size of the infarct area after MI (Pietrasik et al., 2007; Das et al., 2009; Lorgis et al., 2012), which may be responsible for the risk of mortality and MACE in patients with AMI. The risk of cardiovascular mortality was higher than that of all-cause mortality at the time of hospitalization in our meta-analysis. Bozbeyoglu et al. have suggested that cardiovascular mortality associated with fQRS may be due to VA, sudden cardiac death (SCD), and deterioration of left ventricular systolic function secondary to myocardial scarring (Bozbeyoglu et al., 2016). Fragmented QRS is also a predictor of progression to heart failure after MI (Korhonen et al., 2010; Akgul et al., 2015). This meta-analysis correlates heart failure with fQRS, which may lead to an increase in cardiovascular mortality.

It is noteworthy that fQRS had a higher predictive value for hospital mortality than follow-up mortality. Another study showed that the mortality in the fQRS group mainly occurred in the first month after discharge during the follow-up period (Akgul et al., 2015). Therefore, monitoring cardiac rhythm for longer than 24–48 h may be required in patients with fQRS (Attachaipanich and Krittayaphong, 2019), and MI patients with fQRS need more attention during hospitalization and for 1 month after discharge. In particular, fQRS significantly increased in-hospital mortality in the group with *arbitrary ECG*, according to the subgroup analysis. These three studies concluded that fQRS significantly increased the risk of in-hospital mortality, a finding consistent with our observation that one should not ignore the warning signs of transient fQRS found within 48 h of admission. Furthermore, virtually all patients developed fQRS within 48 h after primary PCI, especially within 24 to 48 h (Akbarzadeh et al., 2013; Sheng et al., 2014; Zhang et al., 2017). Therefore, an assessment based on the presence of fQRS on admission ECG may underestimate the predictive value of fQRS for mortality.

### fQRS and MACE

This is the first meta-analysis on the relationship between fQRS and in-hospital MACE in patients with AMI and is consistent with the results of previous studies that established fQRS as a significant predictor for in-hospital MACE in AMI (Yildirim et al., 2014; Bozbeyoglu et al., 2016). Although it is not as high as the value for mortality, this is a significant conclusion that cannot be ignored. Of the five studies included, four studies found a significant correlation between fQRS and MACE events. Stavileci et al. (2014) reported that persistent fQRS on admission ECG was associated with poor prognosis in patients with STEMI. Lorgis et al. (2012) have reported that persistent fQRS and BNP were associated with decreased survival in univariate analysis. However, these were not significant independent predictors of MACE in multivariate Cox regression analysis. This study found that fQRS was not a predictor of heart failure or VA in univariate analysis. Fragmented QRS did not predict in-hospital MACE in STEMI patients in a study by Umapathy et al. (2018), that included STEMI patients presenting Killip I and II with a lower incidence of triple-vessel disease (1.7%) and fQRS resolving in 26% of the patients, indicating better myocardial revascularization (Umapathy et al., 2018). In contrast, other study cohorts included all patients with fQRS who had a higher incidence of triple-vessel disease, larger infarct size, and worse left ventricular function. However, it cannot be ignored that our study only analyzed in-hospital MACE in patients with STEMI, because we did not find any reports of in-hospital MACE in patients with NSTEMI. Therefore, it is unclear whether our findings represent AMI. In terms of long-term MACE prediction, our meta-analysis found that fQRS is closely related to long-term MACE, but that heterogeneity was high, and no obvious abnormality was found in a single study through sensitivity analysis.

### fQRS and Other Cardiovascular Events

Fragmented QRS was an independent predictor of VA in AMI. Attachaipanich *et al*. showed that the time from admission to the last life-threatening arrhythmia event was longer in the fQRS than in the non-fQRS group (Attachaipanich and Krittayaphong, 2019). This meta-analysis found that fQRS was significantly associated with VT events. The impulse conduction velocity delay, reentrant circuit, and increased risk of automaticity due to fibroblast infiltration form an arrhythmogenic substrate associated with an increased risk of cardiac arrhythmia that originates from the peri-infarct area (Attachaipanich and Krittayaphong, 2019). This could also increase the mortality and the risk of MACE.

Overall, we found that fQRS is significantly associated with mortality and MACE in patients with AMI and other adverse events such as heart failure and VA. However, current research is still unclear whether the presence of fQRS leads to a reduction in LVEF and further development of electrophysiological instability, thereby further increasing mortality and events. It has also not yet been established whether the instability of hemodynamics and electrophysiological activities, caused by severe myocardial vascular lesions and larger infarct area, can lead to fQRS and increased risk of MACE and mortality of MI. However, this does not affect the predictive value of fQRS for patient mortality and MACE. The causality is not yet clear, so a series of trials will be needed to prove the cause of MACE and death by excluding other factors such as demographic data, clinical, and laboratory tests on patients at the same level.

### Limitation of This Study

(1) All studies included were observational, and most of them were retrospective. None of the studies evaluated the use of fQRS in a randomized fashion. (2) Long-term cardiovascular follow-up from 5 to 26 months extended over a long time. (3) Fragmented QRS was defined differently among included studies, contributing to the significant heterogeneity found in our meta-analysis. (4) The data extracted were not adjusted for other variables, such as age, past medical history, and ventricular function. Further studies may evaluate whether fQRS can be used as an independent predictor of in-hospital and long-term cardiovascular events in the AMI population. (5) In the exclusion criteria, patients with previous MI and PCI could not be excluded and these patients significantly influenced prognosis and the formation of fQRS.

## Conclusion

The presence of fragmented QRS is associated with increased risk of in-hospital mortality, long-term mortality, and MACE in patients with AMI, while transient fQRS cannot be ignored. Moreover, it is related to an increased risk of in-hospital heart failure and VT/VF events. In the presence of fQRS, the OR for in-hospital mortality is higher than that for long-term mortality. The OR for long-term MACE is higher than that for in-hospital MACE. In terms of the prediction of in-hospital mortality, the predictive value for in-hospital cardiovascular mortality may be higher than that for all-cause mortality. In terms of long-term mortality, however, the predictive value for all-cause mortality may be higher than that for cardiovascular mortality. The in-hospital mortality in the fQRS group was significantly higher than that in the non-fQRS group when only patients with STEMI were included. The long-term mortality in the fQRS group was significantly higher than that in the non-fQRS group when only patients with NSTEMI were included. These results indicate that fQRS has a predictive value for both in-hospital and long-term adverse events. Even though the evidence for RCT trails is limited, it is still a predictor of clinical significance.

## Data Availability Statement

All datasets generated for this study are included in the article.

## Author Contributions

GL and QL participated in the study design, searched databases, extracted, and assessed data. GL drafted the manuscript. YP and ZZ revised the manuscript. GL, QL, and JD extracted data. ZZ designed the study. All authors approved the final version of the manuscript.

## Conflict of Interest

The authors declare that the research was conducted in the absence of any commercial or financial relationships that could be construed as a potential conflict of interest.
