# Supplementary material for: The Predictive Value of Fragmented QRS for Cardiovascular Events in Acute Myocardial Infarction: A Systematic Review and Meta-Analysis
Source: Front Physiol. 2020 Oct 6;11:1027. doi: 10.3389/fphys.2020.01027 (PMC7574772; doi:10.3389/fphys.2020.01027)
Supplement: Supplementary file 1 [file Table_1.DOC]

# **MOOSE Checklist**

# Background

## *Problem definition*

- What is the incidence of fQRS in acute myocardial infarction（AMI）?
- From the existing literature, is there a worldwide consensus for definition and incidence rate (IR) of this condition?
- Are the mortality and adverse events of fQRS consistent with myocardial infarction in different studies?

## *Hypothesis statement*

We propose that differences observed in incidence rate of fQRS across studies are likely to be due to differences in case ascertainment , inclusion criteria and study methodology but may be influenced by individual factors as well.

## *Study outcomes*

The prespecified outcomes were mortality and Major Adverse Cardiovascular Events during in-hospital or follow-up.

## *Type of exposure or intervention used*

- No exposure or intervention was tested in the studies included in this review;
- Cases of AMI were studied and observed characteristics recorded;
- Comparison of clinical characteristics and incidence of adverse events between fQRS group and non-fQRS group in patients with AMI.

## *Type of study design used*

- Studies meeting all of following criteria were eligibility: Cohort studies (prospective or retrospective), cross-sectional studies, randomised control trials reported in-hospital mortality /MACE or long-term mortality/MACE in AMI patients with and without fQRS.

## *Study population*

- Patients included in individual studies were diagnosed with acute myocardial infarction according to accepted clinical criteria.
- Studies including only patients with AMI, non-ST segment elevation myocardial infarction (NSTEMI)and ST-segment elevation myocardial infarction(STEMI), and the experimental group and control groups were fQRS group and Non-fQRS group, respectively, with at least one endpoint event.

# Reporting of search strategy

## *Qualifications of searchers*

- Mr Gongming Luo was trained in methods of literature searching as part of his Master's degree studies, Lanzhou University;
- The selection of documents were completed by Mr. Luo, Ms. Li, and the controversial documents were decided by the third decision-maker, Professor Zhang Zheng;
- Search methodology was discussed with Zheng Zhang, Professional, Lanzhou university.

##### Search strategy

- Two researchers independently searched the Embase, Cochrane-Library, Medline and PubMed databases for published studies from January 2010 to March 2020, using a search strategy that including the terms "fragmented QRS ", "acute myocardial infarction", "STEMI", "NSTEMI", "MACE", "cardiovascular events" and "mortality". Only full articles in English and studies conducted were included.

## *Effort to include all available studies*

- The final list of included studies was discussed with experts in the field (Professor Zheng Zhang Cardiovascular Group, the First Hospital, Lanzhou University.CHINA)
- Studies in English languages were included
- Authors were not contacted, as adequate information for the performance of this review was available from studies and abstracts.

## *Databases and registries searched*

- Embase and Medline (January 2010 -March 2020) = 199 hits
- Pubmed (– March 2020) = 131hits.
- Cochrane (– March 2020) = 13hits
- Web of science (January 2010– March 2020) = 240 hits

All: 583 hits

## *Search software used*

- PubMed was accessed from the National Library of Medicine (free online)
- Medline and Embase are available on the OVID SP platform
- Web of Science is available on Web Science Group platform
- Cochrane Library is available on the Wiley Interscience platform.

## *Use of hand searching*

- The bibliography of all included studies and those of previous reviews on the subject were examined for further relevant studies

## *List of citations located and those excluded*

- Following the above search strategy, the total initial studies were identified from four online databases. After 193 duplicates were removed, we screened the remaining 102 articles by reading title and abstract.
- 102 articles were excluded because they did not meet the inclusion criteria.
- 148 studies were removed after a total of 172 articles were screened by reading the full text.
- We found that the observational endpoints set by three studies did not match our preset observational endpoints when we compared the endpoint events in the eligible studies.
- We excluded an article because the sample was the same as the included study.
- [20](javascript:;) studies were available included in this meta-analysis (***Figure 1***).

## *Inclusion and exclusion criteria*

| **Inclusion criteria** | **Exclusion criteria** |
| --- | --- |
| Cohort studies (prospective or retrospective)  cross-sectional studies  randomised control trials | Case series Non-English articles |
| Reported in-hospital mortality /MACE or long-term mortality/MACE in AMI patients with and without fQRS. | Exclude studies with no pre-defined end point events |
| Studies including only patients with AMI, non-ST segment elevation myocardial infarction (NSTEMI)and ST-segment elevation myocardial infarction(STEMI), and the experimental group and control groups were fQRS group and Non-fQRS group, respectively, with at least one endpoint event. | Otherwise, the research will be excluded |

- Excluded papers are listed in Figure1 with reason for exclusion for all those read in full.

## *Non-English articles*

Exclude all.

## *Methods of handling abstracts and unpublished data*

- We excluded proceedings papers and assessed them for eligibility according to our inclusion and exclusion criteria.
- 1 handling abstract papers were included. The abstracts provided adequate information for the purposes of this review so the authors were not contacted.
- As stated previously, the list of included studies was discussed with experts in the field and deemed to be complete. Beyond this and identification of unpublished papers we did not attempt to identify as yet unpublished data.

## *Description of contact with authors*

- Contact was not made with authors of the studies.

# Reporting of methods

## *Description of relevance or appropriateness of studies assembled for assessing the hypothesis to be tested*

- All studies provide observational epidemiological data on patients with confirmed diagnoses of AMI.
- All continents are represented except for Australia and America.
- Studied period spans from 2010 to 2020.
- Study methodology is well documented
- This allows analysis of rates by inclusion criteria, different diseases (STEMI or NSTEMI), end-event grouping (cardiovascular death and all-cause death), and study type in order to test the hypothesis of the influence of these factors on the observed rates.

## *Rationale for selection and coding of data*

- Studies were included and excluded as per criteria outlined above
- Incidence rates and cardiovascular events rates between fQRS and non-fQRS in AMI and 95% confidence intervals were calculated according to the Poisson distribution by hand from crude data extracted.
- Year was documented as final-year of study
- Where available, age and sex incidence figure were also recorded and age/sex specific incidence rates calculated.

## *Data extraction proforma*

| Title |  | | | | |
| --- | --- | --- | --- | --- | --- |
| **Reference** |  | | | | |
| **Authors** |  | | | | |
| **Country/Area** |  | | | | |
| **Inclusion criteria** |  | | | | |
| **Exclusion criteria** |  | | | | |
| **Definition of fQRS** |  | | | | |
| **Population studied** |  | | | | |
| **No. of cases** |  | | | | |
| **Conclusion** |  | | | | |
| **In-hospital Mortality** |  | **C.I.** |  | **OR** |  |
| **In-hospital MACE** |  | **C.I.** |  | **OR.** |  |
| **Long-term Mortality** |  | **C.I.** |  | **OR.** |  |
| **Long-term MACE** |  | **C.I.** |  | **OR.** |  |
| **Notes** |  | | | | |

## *Documentation of how data were classified/coded*

- After data extraction and calculation of individual study estimates. 5 studies were selected at random (using a random numbers table) and data extraction validated by JMcC (blindly) with full agreement.

## *Assessment of confounding*

- Not applicable

## *Assessment of study quality*

- The internal validity of studies was assessed using the Quality in Prognosis Studies (QUIPS) tool.
- A Funnel plot and the Egger test were used to test for any potential publication bias.

## *Criteria for assessment of study quality*

### High Risk

## The relationship between the prognostic factors(PF) and outcome is very likely to be different for participants and eligible nonparticipants

AND/OR

The relationship between the PF and outcome is very likely to be different for completing and non-completing participants.

AND/OR

The measurement of the PF is very likely to be different for different levels of the outcome of interest.

AND/OR

The measurement of the outcome is very likely to be different related to the baseline level of the PF rest.

### Intermediate Risk

### The relationship between the PF and outcome may be different for participants and eligible nonparticipants

## AND/OR

# The relationship between the PF and outcome may be different for completing and non-completing participants

## AND/OR

The measurement of the PF may be different for different levels of the outcome of interest

## AND/OR

The measurement of the outcome may be different related to the baseline level of the PF rest.

### Low Risk

### The relationship between the PF and outcome is unlikely to be different for participants and eligible nonparticipants

## AND/OR

### The relationship between the PF and outcome is unlikely to be different for completing and noncompeting participants

## AND/OR

### The measurement of the PF is unlikely to be different for different levels of the outcome of interest

## AND/OR

### The measurement of the outcome is unlikely to be differently related to the baseline level of the PF

## *Assessment of heterogeneity*

- The hypothesis stated that any endpoints differences in rates might be related to variation in Type of disease, inclusion criteria and type of study. This was tested by looking for a significant reduction in heterogeneity by Sensitivity analysis and Meta-regression analysis in these ways
- Using Stata statistical package heterogeneity was assessed using Q and I^2^ statistics for OR.
- Studies were then grouped according to type study, inclusion criteria, difference endpoints (cardiovascular and all-cause death) and according to STEMI and NSTEMI and heterogeneity calculated for each subgroup.
- The sensitivity analysis was performed by calculating the number of patients in the trial as a percentage of the total number of analysed to value the weight of the overall results of the meta-analysis for each study.
- Meta-regression analysis was used to assess the potential impact of baseline characteristics on heterogeneity.

## *Description of statistical methods*

- Statistical analysis was performed using Review Manager 5.1, State 16, and Meta-Disc 1.4 software.
- We extracted the baseline data and end events of the experimental group and the control group in each study. For the baseline data, the counting data used the chi-square test, and the continuity data used the t-test after summarising the data.
- The difference was statistically significant with p<0.05. Meta-analysis was performed according to different endpoint events, and we also conducted a subgroup analysis to evaluate NSTEMI and STEMI, inclusion criteria, and, more importantly, cardiovascular mortality and all-cause mortality, respectively.
- The effect size was presented as the odds ratio(OR), likelihood ratio (LR) indicating how many times more (or less) likely a patient experiencing an endpoint is to express fQRS.
- Since heterogeneity in endpoints from study to study was anticipated, a random effect model was used for the primary analysis with study as random variable.
- Heterogeneity was assessed the *I^2^* statistic test and its 95% confidence intervals (CI) and the random-effects model was used to account for significant statistical variation; A P value of<0.05 was considered to be statistically significant.
- The sensitivity analysis was performed by calculating the number of patients in the trial as a percentage of the total number of analysed to value the weight of the overall results of the meta-analysis for each study.
- Meta-regression analysis was used to assess the potential impact of baseline characteristics on heterogeneity.

# Reporting of results

## *Graphic summarising of individual study estimates and pooled estimate*

- Forest plots are drawn on the integer scale for ease of interpretation
- The effect size was presented as the odds ratio(OR), likelihood ratio (LR) indicating how many times more (or less) likely a patient experiencing an endpoint is to express fQRS.
- Studies with greater heterogeneity reduce these heterogeneities through meta-regression analysis and sensitivity analysis.
- The results of different factors of the same endpoint event are reported separately (Table4)

##### Indication of statistical uncertainty of findings

95% confidence intervals (according to the Poisson distribution) for all calculated rates are given

# Reporting of discussion

## *Quantification of publication bias*

- Using funnel plots and Eger's tests assessed publication bias.

## *Justification of exclusion*

- Papers were excluded on the basis of exclusion criteria listed.

##### Assessment of quality of included studies

The methodological quality of the included studies was generally good, without high risk of bias (Table 1). Three studies have different inclusion criteria for fQRS, and therefore the conclusions may be bias compared with other studies. Three studies did not give precise reason concerning the loss to follow-up, and consequently, the relationship between fQRS and outcome may be different for completing and non-completing participants. The funnel plot and Egger test did not suggest evidence of publication bias (***Figure 2 and figure 3***).

# Reporting of conclusions

## *Consideration of alternative explanations for observed results*

- See discussion

## *Generalisation of these conclusions*

- See discussion

## *Guidelines for future research*

- See discussion

## *Disclosure of funding source*

- None.
